# Gene expression and ultra-structural evidence for metabolic derangement in the primary mitral regurgitation heart

**DOI:** 10.1093/ehjopen/oeae034

**Published:** 2024-05-01

**Authors:** Mariame Selma Kane, Juan Xavier Masjoan Juncos, Shajer Manzoor, Maximiliano Grenett, Joo-Yeun Oh, Betty Pat, Mustafa I Ahmed, Clifton Lewis, James E Davies, Thomas S Denney, Jonathan McConathy, Louis J Dell’Italia

**Affiliations:** Division of Cardiovascular Disease, Heersink School of Medicine, University of Alabama at Birmingham (UAB), 1900 University Boulevard, Birmingham, AL 35233, USA; Birmingham Veterans Affairs Health Care System, 700 South 19th Street, Birmingham, AL 35233, USA; Division of Cardiovascular Disease, Heersink School of Medicine, University of Alabama at Birmingham (UAB), 1900 University Boulevard, Birmingham, AL 35233, USA; Division of Cardiovascular Disease, Heersink School of Medicine, University of Alabama at Birmingham (UAB), 1900 University Boulevard, Birmingham, AL 35233, USA; Division of Cardiovascular Disease, Heersink School of Medicine, University of Alabama at Birmingham (UAB), 1900 University Boulevard, Birmingham, AL 35233, USA; Division of Cardiovascular Disease, Heersink School of Medicine, University of Alabama at Birmingham (UAB), 1900 University Boulevard, Birmingham, AL 35233, USA; Birmingham Veterans Affairs Health Care System, 700 South 19th Street, Birmingham, AL 35233, USA; Division of Cardiovascular Disease, Heersink School of Medicine, University of Alabama at Birmingham (UAB), 1900 University Boulevard, Birmingham, AL 35233, USA; Birmingham Veterans Affairs Health Care System, 700 South 19th Street, Birmingham, AL 35233, USA; Division of Cardiovascular Disease, Heersink School of Medicine, University of Alabama at Birmingham (UAB), 1900 University Boulevard, Birmingham, AL 35233, USA; Division of Thoracic and Cardiovascular Surgery, Department of Surgery, University of Alabama at Birmingham (UAB), 1808 7th Avenue, Birmingham, AL 35294, USA; Division of Thoracic and Cardiovascular Surgery, Department of Surgery, University of Alabama at Birmingham (UAB), 1808 7th Avenue, Birmingham, AL 35294, USA; Division of Thoracic and Cardiovascular Surgery, Department of Surgery, University of Alabama at Birmingham (UAB), 1808 7th Avenue, Birmingham, AL 35294, USA; Samuel Ginn College of Engineering, Auburn University, 345 W Magnolia Ave, Auburn, AL 36849, USA; Department of Radiology, University of Albama (UAB), 619 19th Street South, Birmingham, AL 35294, USA; Division of Cardiovascular Disease, Heersink School of Medicine, University of Alabama at Birmingham (UAB), 1900 University Boulevard, Birmingham, AL 35233, USA; Birmingham Veterans Affairs Health Care System, 700 South 19th Street, Birmingham, AL 35233, USA

**Keywords:** Primary mitral regurgitation, [^18^F]FDG-PET/CMR, Lipid and glucose metabolism, Mitochondria dysfunction

## Abstract

**Aims:**

Chronic neurohormonal activation and haemodynamic load cause derangement in the utilization of the myocardial substrate. In this study, we test the hypothesis that the primary mitral regurgitation (PMR) heart shows an altered metabolic gene profile and cardiac ultra-structure consistent with decreased fatty acid and glucose metabolism despite a left ventricular ejection fraction (LVEF) > 60%.

**Methods and results:**

Metabolic gene expression in right atrial (RA), left atrial (LA), and left ventricular (LV) biopsies from donor hearts (*n* = 10) and from patients with moderate-to-severe PMR (*n* = 11) at surgery showed decreased mRNA glucose transporter type 4 (GLUT4), GLUT1, and insulin receptor substrate 2 and increased mRNA hexokinase 2, O-linked *N*-acetylglucosamine transferase, and O-linked *N*-acetylglucosaminyl transferase, rate-limiting steps in the hexosamine biosynthetic pathway. Pericardial fluid levels of neuropeptide Y were four-fold higher than simultaneous plasma, indicative of increased sympathetic drive. Quantitative transmission electron microscopy showed glycogen accumulation, glycophagy, increased lipid droplets (LDs), and mitochondrial cristae lysis. These findings are associated with increased mRNA for glycogen synthase kinase 3β, decreased carnitine palmitoyl transferase 2, and fatty acid synthase in PMR vs. normals. Cardiac magnetic resonance and positron emission tomography for 2-deoxy-2-[^18^F]fluoro-D-glucose ([^18^F]FDG) uptake showed decreased LV [^18^F]FDG uptake and increased plasma haemoglobin A1C, free fatty acids, and mitochondrial damage-associated molecular patterns in a separate cohort of patients with stable moderate PMR with an LVEF > 60% (*n* = 8) vs. normal controls (*n* = 8).

**Conclusion:**

The PMR heart has a global ultra-structural and metabolic gene expression pattern of decreased glucose uptake along with increased glycogen and LDs. Further studies must determine whether this presentation is an adaptation or maladaptation in the PMR heart in the clinical evaluation of PMR.

## Graphical Abstract

**Graphical Abstract:**
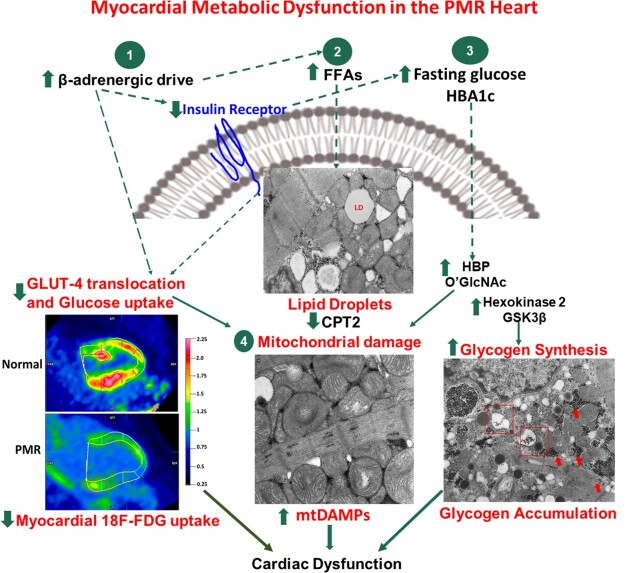
Chronic β-adrenergic drive causes: (i) insulin receptor inhibition, decreased glucose transporter 4, and glucose (red) uptake; (ii) increased free fatty acids and lipid droplets; (iii) increased hexosamine biosynthetic pathway/O-linked *N*-acetylglucosaminyl and glycogen accumulation; and (iv) mitochondrial damage, increased mitochondrial damage-associated molecular patterns and inflammation, and cardiac dysfunction.

## Introduction

Chronic primary mitral regurgitation (PMR) results in a gradual progression of left atrial (LA) and left ventricular (LV) dilatation that can result in heart failure, arrhythmias, and sudden death.^1^ There is a gap in knowledge regarding the optimal timing for surgical correction in asymptomatic patients with PMR because there is a 20% chance of such patients having an left ventricular ejection fraction (LVEF) < 50% after mitral valve repair despite having an LVEF > 60% prior to surgery.^2^ We have demonstrated severe cardiomyocyte cytoskeletal breakdown, myolysis, oxidative stress, and mitochondrial damage in patients with PMR.^3–6^ The underlying cause of the cardiomyocyte ultra-structural damage can be attributed to the well-documented increase in adrenergic drive in the PMR heart.^7–11^ Previous studies have established an association between sustained beta-adrenergic receptor (βAR) stimulation and increased sympathetic nervous activity and insulin resistance in the heart.^12–15^

Insulin resistance is a condition in which cells have a defective glucose uptake in response to insulin. Sustained β_1_AR stimulation results in the inhibition of insulin-induced glucose uptake and a reduction of glucose transporter 4 (GLUT4) expression in cardiomyocytes.^16–18^ Patients with severe heart failure exhibit insulin resistance and the degree of insulin resistance correlates with the severity of heart failure.^19^ Studies in transgenic mice with a cardiac-specific overexpression of G protein-couple receptor kinase 2 (GRK2) demonstrate a desensitization of insulin signalling and decreased 2-deoxy-2-[^18^F]fluoro-D-glucose ([^18^F]FDG) uptake, which increases after ischaemic injury and precedes the development of heart failure.^20^ In patients with heart failure, impaired insulin signalling and accumulation of lipotoxic species are reversible after a period of mechanical unloading with a long-term LV assist device.^21^ It is postulated that the removal of sustained adrenergic drive in the unloaded LV is a potential reversible mechanism behind the accumulation of toxic and neutral lipid species in the myocardium.^13^

We have previously reported insulin resistance and increased adrenergic drive in a microarray analysis of LV biopsies from patients free of epicardial coronary artery disease and with moderate-to-severe PMR and an LVEF > 60%.^7^ In this gene array, we show an up-regulation of cAMP and G-protein-coupled signalling and decreased insulin receptor signalling, suggesting an insulin resistance-like metabolic derangement. In addition, we have reported mitochondrial cristae lysis, increased lipid droplets (LDs), increased glycogen accumulation, and glycophagy in both LA and LV by transmission electron microscopy (TEM) in patients with PMR.^3–6^ In the present study, we test the hypothesis that biopsies in patients with surgical PMR have a myocardial metabolic gene profile that matches these cardiomyocyte ultra-structural changes, by using a panel of metabolic genes previously identified from our gene array.^7^ In a subset of patients with stable PMR, we also perform cardiac magnetic resonance (CMR) and positron emission tomography (PET) to measure [^18^F]FDG uptake in a separate cohort of patients with PMR with an LVEF > 60% prior to surgery.

## Methods

### Patients with primary mitral regurgitation and controls for metabolic gene expression

The study population included 11 patients with moderate-to-severe PMR by echo/Doppler secondary to degenerative mitral valve disease, referred for corrective mitral valve surgery (*Table 1*). All patients had cardiac catheterization before surgery and were excluded for obstructive coronary artery disease (>50% stenosis), aortic valve disease, or mitral stenosis. Normal LV, LA, and right atrial (RA) tissue (*n* = 10; 60% males; 60% Whites) was obtained from non-failing human hearts rejected for transplantation (Duke Human Heart Repository, Duke University, Durham, NC, USA). Patient recruitment occurred between 2015 and 2019 under the NHLBI Program Project Grant National Institutes of Health Grant (P01 HL051952). Blood and pericardial fluid (PCF) were collected at the time of opening the pericardial sac during mitral valve repair/replacement surgery. The protocol was approved by the University of Alabama at Birmingham Institutional Review Board, and written informed consent was obtained from all participants.

**Table 1:** Tissue biopsies for gene analysis

	Normal (*n* = 10)	PMR (*n* = 11)
Age (years)	52 ± 11	64 ± 10
Sex		
Female	4	5
Male	6	6
Race		
Black	4	9
White	6	2
Other		
Body mass index	24.3 ± 6.6	31.2 ± 3.8
Hypertension	6	9 (73%)
Atrial fibrillation	0	2 (18%)
Type II diabetes	0	3 (27%)
Medications		
None		1 (9%)
Beta-blocker		9 (82%)
ACE inhibitor		1 (8%)
AT_1_RB		5 (45%)
Calcium entry blocker		3 (27%)
Diuretics		4 (3%)
Statins		7 (64%)
Anti-arrhythmia		3 (27%)
Metformin		3 (27%)
NYHA functional class		
I		1 (9%)
II		5 (45%)
III		5 (45%)
Echo/Doppler		
LV end-diastolic dimension (mm)		52 ± 7
LV end-systolic dimension (mm)		35 ± 8
LV ejection fraction (%)		57 ± 8
LA dimension (mm)		46 ± 1
PA systolic pressure (mmHg)		40 ± 7
TR none/moderate/severe		3/7/1
Haematocrit (%)		41 ± 4

Values: mean ± SD or *n* (% of total).

### Patients with primary mitral regurgitation and controls for 2-deoxy-2-[^18^F]fluoro-D-glucose-positron emission tomography/cardiac magnetic resonance

In a separate cohort, eight patients with moderate-to-severe PMR and normal controls with no history of cardiovascular disease underwent [^18^F]FDG-PET/CMR imaging. Patients were recruited during their visit to the UAB Structural Heart Disease Clinic during their clinical care follow-up. Patients with PMR diagnosis not undergoing surgery were selected for the study. All protocols were approved by the University of Alabama at Birmingham Institutional Review Board, and written informed consent was obtained from all participants. The exclusion criteria were as follows: ≤18 years’ old, pregnancy, known history of coronary artery disease, coronary revascularization, myocardial infarction, congestive heart failure, non-ischaemic cardiomyopathy, diabetes mellitus, chronic kidney disease with a glomerular filtration rate <60 mL/min/1.73 m^2^, mitral stenosis, aortic stenosis, >mild aortic regurgitation, >mild tricuspid regurgitation, and cancer with exposure to chemotherapy/radiotherapy.

### Positron emission tomography imaging

Simultaneous with CMR, [^18^F]FDG-PET cardiac imaging on a hybrid PET/CMR system was performed (GE Signa PET-MR, 3T, Chicago, IL, USA). Image acquisition was optimized for entire heart volume, including LA and RA, as described in our laboratory.^3–6^ Cardiac magnetic resonance comprised electrocardiogram-gated sequences, including balanced steady-state free-precision cine images (two-chamber, three-chamber, four-chamber, and complete short-axis stack of LV and LA). Quantification of left ventricle (LV) and right ventricle (RV) function, volumes, and mass was performed using the Qmass module (Medis 8.1, Leiden, The Netherlands).

Patients were studied after an 18 h fast or high–fat, low-carbohydrate diet. A standard clinical dosage of 370 MBq (10 mCi) [^18^F]FDG (PETNET Solutions Inc., Birmingham, AL, USA) was then injected intravenously, in a volume of ≤10 mL over ∼1 min, followed by a saline flush. After [^18^F]FDG injection, the participants rested quietly with minimal movement for 50 min within the PET/CMR suite. Approximately 50 min after [^18^F]FDG injection, the patients were positioned in the PET/CMR scanner for a simultaneous PET and CMR scanning. The initial set of images began at ∼60–70 min after [^18^F]FDG injection and consisted of thoracic field acquisition centred over the heart using T2 images as a reference point. The PET data were reconstructed with ordered subset expectation maximization per manufacturer recommendations with Dixon-based magnetic resonance (MR)-based attenuation correction.

### Positron emission tomography analysis

Fusion and quantification of the PET and CMR data were performed by using the commercially available software package cardiac MIM Encore (MIM Software Inc., Cleveland, OH, USA). Positron emission tomography quantification was performed on [^18^F]FDG-PET data sets by drawing regions of interest over the LV myocardium and calculating the mean standard uptake (SUV_mean_) and maximum SUV (SUV_max_) alone and corrected for intraventricular blood concentration and liver uptake. Body weight was used for the SUV calculations. The patients were evaluated for the presence, amount, and pattern of [^18^F]FDG uptake using a 17-segment American Heart Association (AHA) model at the LV base, LV mid wall, and LV apex.^22^

### Transmission electron microscopy

Tissue was prepared as previously described^6^ to assess mitochondrial cristae integrity, glycogen accumulation, and LD number. Transmission electron microscopy analyses assessed mitochondrial cristae integrity, glycogen accumulation, and LD number in cardiac biopsies of patients with PMR. Images were acquired using two magnifications: ×5000 for lipid number counting and glycogen accumulation and ×20 000 for mitochondrial cristae integrity assessment. All parameters were assessed blindly using the National Institutes of Health Image J software. The total glycogen analysis consisted of determining the total area of the image consisting of glycogen (glycogen area) using the single-particle tool in Image J. Cristae integrity was assessed double blinded using a scoring system of 1–3, with a score of 1 representing 90% of the field having perfect mitochondria with tightly packed cristae, a score of 2 representing 70–50% of mitochondria having slightly disorganized cristae, and finally, a score of 3 representing <30% of mitochondria denoting normal cristae and a majority of mitochondria having completely disorganized cristae, with swollen white space (empty mitochondria).

### Plasma and pericardial fluid biomarkers and mitochondrial damage–associated molecular patterns

Mitochondrial (mt) DNA damage–associated molecular patterns (DAMPs) and other biomarkers were assessed in cell-free plasma (150 µL) and from PCF^23^ collected from patients with PMR (2.5 μL of the MagMax Cell-Free DNA Magnetic Beads per sample). Cell-free DNA was eluted in 20 μL volumes, and mtDNA DAMPs were assessed via amplification of DNA within the nicotinamide adenine dinucleotide + hydrogen (NADH) dehydrogenase Subunit 1 and NADH dehydrogenase Subunit 6 regions of the mtDNA by real-time polymerase chain reaction (PCR) using the CFX Opus 384 Real-Time PCR System (Bio-Rad Laboratories, Hercules, CA, USA). Mitochondrial DNA DAMP copies were quantified relative to standard samples of known copies (10–100 000 copies). Data are expressed as mtDNA DAMPs per microliter of plasma.^24^

Neat plasma and/or PCF neuropeptide Y (NPY), free fatty acid (FFA), glucose-6-phosphate dehydrogenase, and apolipoprotein A1 (ApoA1; samples diluted 1:50 000) were all measured using commercially available kits, according to the manufacturer’s instruction: Human NPY/NPY DuoSet ELISA (#DY8517-05; R&D Systems Inc., Minneapolis, MN, USA), Free Fatty Acid Assay Kit—Quantification (#ab65341; Abcam, Waltham, MA, USA), Glucose 6 Phosphate Dehydrogenase Assay Kit (Colorimetric) (#ab102529; Abcam), and Human ApoA1 ELISA Kit (#EKE60115-96T; Biomatik, Wilmington, DE, USA). Mitochondrial DNA DAMPs were assayed in 150 μL of cell-free PCF using the same method for plasma (see above). Paired plasma and PCF samples from the same patient were run (in duplicate) simultaneously for all measurements. All assays were performed in triplicate and tested to ensure that samples fell within the optimal standard range of detection.

### RNA isolation and real-time reverse transcription polymerase chain reaction

Total RNA was extracted from LV biopsies using Qiagen RNeasy Fibrous Tissue Mini Kit (Qiagen Sciences, Germantown, MD, USA) and cleaned by Ambion TURBO™ DNase (Thermo Fisher Scientific). Ten normal non-failing human RNA samples were extracted from LA, RA, and LV tissue from motor vehicle accident subjects (Duke Human Heart Repository, Duke University, Durham, NC, USA). Reverse transcription was performed using the High-Capacity cDNA Reverse Transcription kit (Applied Biosystems, Carlsbad, CA, USA). Real-time PCR was performed using the CFX Opus 384 Real-Time PCR System (Bio-Rad Laboratories) with Taqman Gene Expression assays containing gene-specific primers and probes (Thermo Fisher Scientific; see Supplementary material online, *Table S1*). Relative gene expression was determined using the comparative cycle threshold method. Data were normalized to 18S and expressed as a fold change from normal non-failing subjects.

### Statistical analysis

Results are expressed as mean ± standard error of the mean unless otherwise indicated. Statistical analyses were performed using GraphPad Prism 10.0.2. Data were reviewed for normality using QQ plot and Shapiro test prior to statistical analysis. Data points outside 2 SDs from the mean were excluded from the statistical analyses. Student’s *t*-test was used for pair-wise comparisons, and Kolmogorov–Smirnov test was performed to compare cumulative distributions. An analysis of variance was used for multiple group comparison with test the null hypothesis that all samples were drawn from a single population and if significant differences existed (*P* < 0.05). Tests were followed by Tukey’s *post hoc* test. The correction for multiple comparisons was evaluated by Sidak.

## Results

### Patient demographics and cardiac magnetic resonance function

*Table 1* lists demographics in patients with PMR who had heart biopsy and their respective normal groups. Males and females were nearly equally distributed; however, patients with PMR had a higher body mass index. Six of 10 normal and 9 of 11 had hypertension based on the list of medications in the PMR group. There were five Class III, five Class II, and only one Class I NYHA (New York Heart Association) PMR patients. Mean LV end-systolic dimension is 35 mm and mean LVEF is 58% with 10 of 11 PMR patients with an LVEF > 55%. Of note, three patients with PMR had non-insulin-dependent diabetes mellitus. Plasma NPY (3.07 ± 4.30 vs. 1.96 ± 1.10 ng/mL *P* = 0.03) and FFAs (382 ± 168 vs. 154 ± 45 µM, *P* = 0.003) were higher than normal, and ApoA1 (0.93 ± 0.50 vs. 1.69 ± 0.89 mg/mL, *P* = 0.01) was lower than normals.

### Lipid metabolism and lipid droplet accumulation in primary mitral regurgitation

#### Left ventricle

**Figure 1:**
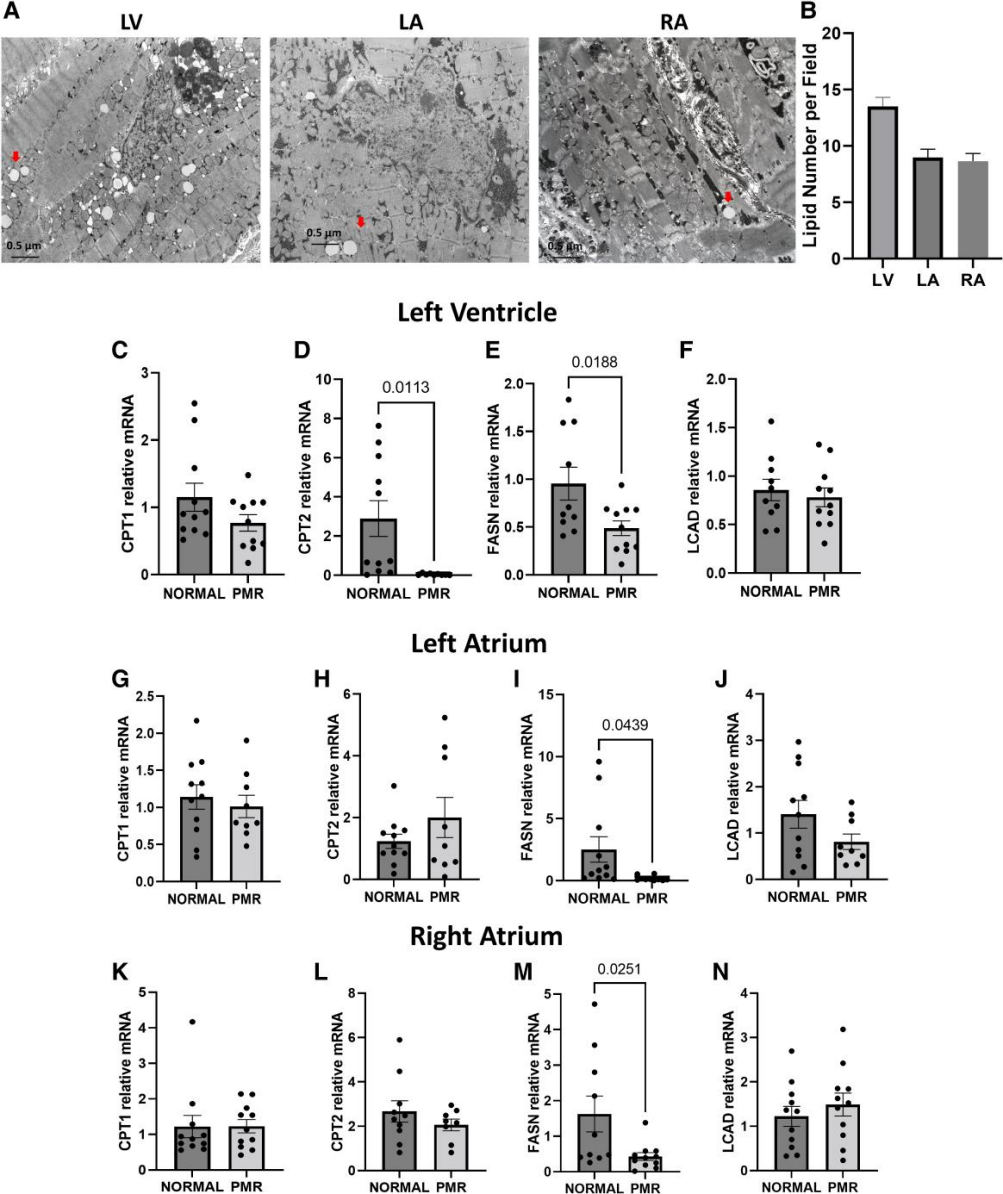
Lipid metabolism. (*A* and *B*) Lipid droplets in primary mitral regurgitation cardiac tissues. (*A*) Representative transmission electron microscopy image of the left ventricle, right atrium, and left atrium from human cardiac tissue biopsies. The arrows show lipid droplets. (*B*) Total lipid droplets number in one field (magnification, ×5000) were assessed double blindly. Data were inferred from 10 to 15 fields for each patient (*n* = 6–10). (*D–N*) Gene expression of lipid metabolism from the hearts of patients with normal and primary mitral regurgitation. Data normalized to 18S and expressed as a fold change from normal (*n*= 8–11 subjects/group). Data presented as mean ± standard error of the mean. *P*-values indicated on graph. Multiple comparison adjustment was performed when comparing more than three groups. CPT, carnitine palmitoyltransferase 1 and 2; FASN, fatty acid synthase; LCAD, long-chain acyl-CoA.

Quantitative TEM of tissue biopsies of patients with PMR demonstrates increased LDs (with arrows) in LV in particular (*Figure 1A* and *B*). Tissue biopsies demonstrate decreased expression of genes involved in lipid metabolism; particularly, carnitine palmotyl transferase (CPT) 2 and fatty acid synthase (FASN) in LV PMR vs. normal (*Figure 1D–F*).

#### Left atrium and right atrium

Transmission electron microscopy images of RA and LA show the presence of LDs. However, the RA and LA show no alteration in CPT1 and CPT2 mRNA (*Figure 1G*, *H* and *K*, *L*), while FASN mRNA is decreased in both LA and RA (*Figure 1M* and *N*).

**Figure 2:**
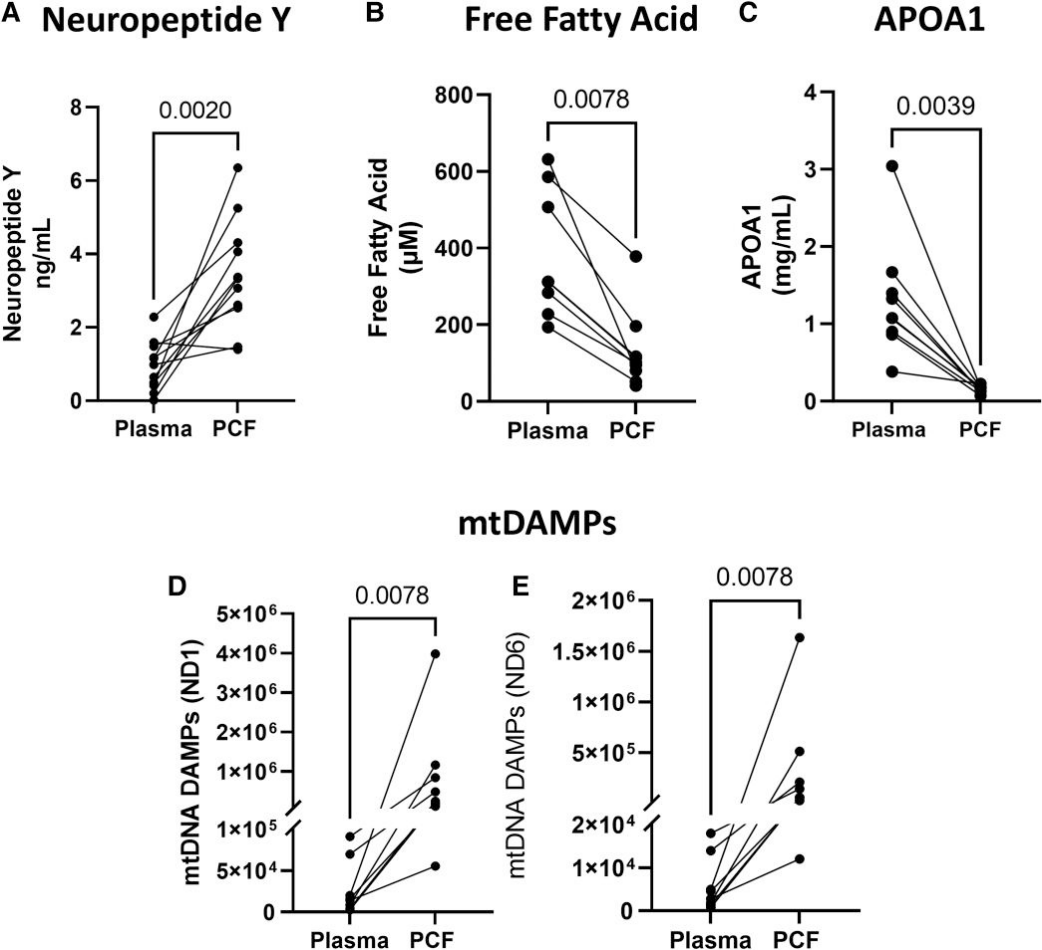
Plasma markers of adrenergic drive and lipid derangement in patients with primary mitral regurgitation. (*A*) Neuropeptide Y, (*B*) free fatty acids, (*C*) apolipoprotein A1, and (*D* and *E*) mitochondrial damage-associated molecular patterns from the ND1 and ND6 regions, respectively, were measured in both plasma and pericardial fluid of patients with primary mitral regurgitation (*n* = 11). *P*-values indicated on the graph.

To further investigate altered lipid metabolism in PMR, we assessed myocardial FFA and ApoA1 levels in PCF vs. plasma (*Figure 2*). Both FFA and ApoA1 are significantly decreased in the PCF vs. plasma of PMR patients (*Figure 2B* and *C*). In addition, NPY levels are significantly higher in PCF vs. plasma of PMR patients (*Figure 2A*).

### Mitochondrial damage in primary mitral regurgitation

**Figure 3:**
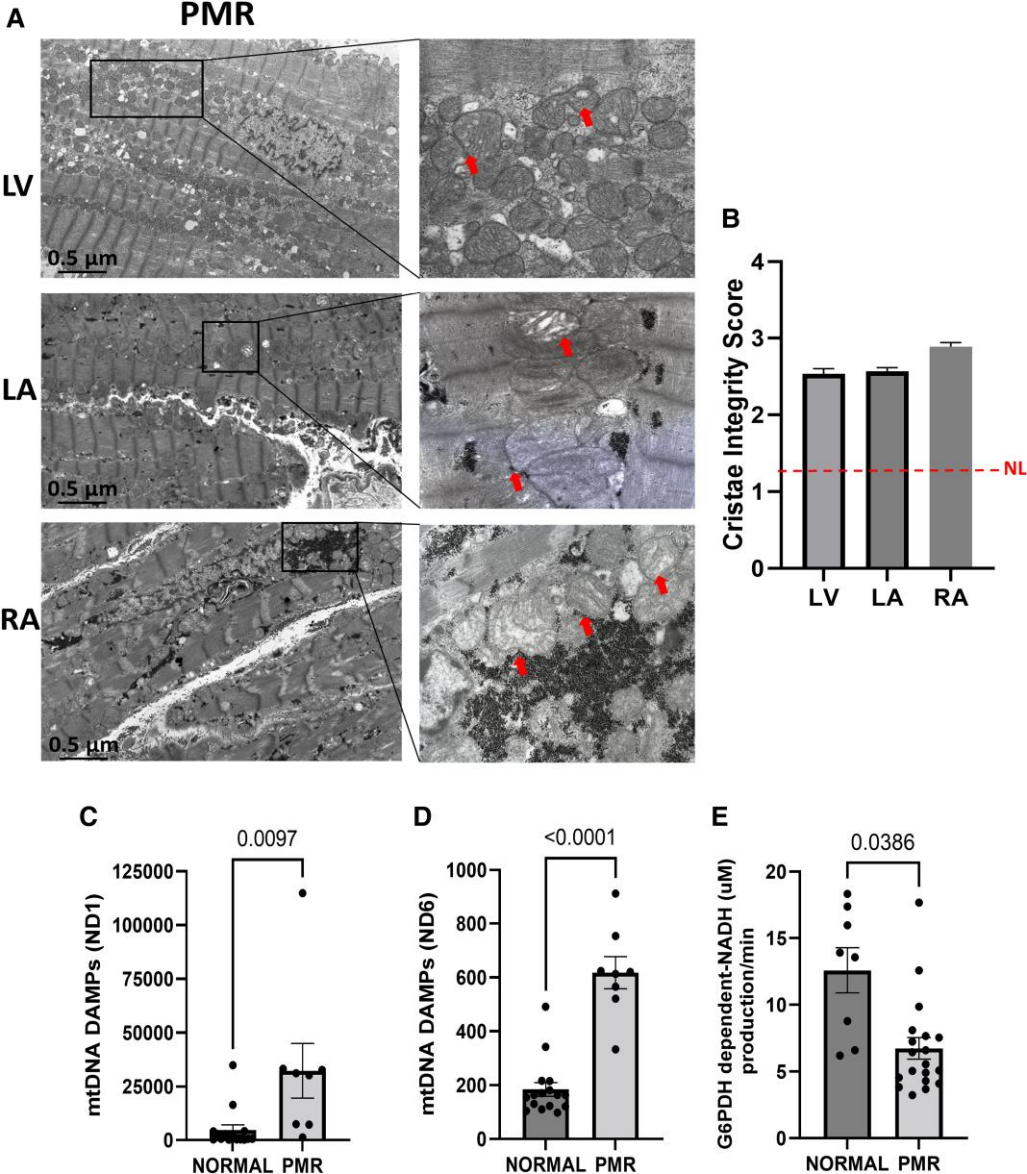
Mitochondrial damage in patients with primary mitral regurgitation. (*A* and *B*) Quantitative transmission electron microscopy analysis of mitochondrial ultra-structure in primary mitral regurgitation hearts biopsies. (*A*) Representative transmission electron microscopy image of the left ventricle, left atrium, and right atrium (zoom areas are boxed), and the arrows show disrupted mitochondrial cristae. (*B*) Cristae integrity: normal cristae (=1) and complete disorganized cristae (=3). Cristae integrity assessment was performed double blindly using the scoring level. Data were inferred from 10 fields for each heart region from 6 to 10 patients with primary mitral regurgitation. (*C* and *D*) Plasma mitochondrial DNA damage-associated molecular pattern levels of patients with normal and primary mitral regurgitation from the ND1 and ND6 regions, respectively. (*E*) Glucose-6-phosphate dehydrogenase–dependent NADH production/min in the plasma of patients with normal and severe primary mitral regurgitation plasma (*n* = 8–16). Data are presented as mean ± standard error.

Transmission electron microscopy images show extensive mitochondrial damage with substantial cristae disruption (red arrows, *Figure 3*) in RA, LA, and LV (*Figure 3A* and *B*). In addition, mtDNA DAMP plasma levels within the NADH dehydrogenase Subunit 1 and NADH dehydrogenase Subunit 6 regions are higher in PCF vs. plasma supporting a myocardial source (*Figure 2D*). Mitochondrial damage and circulating mtDNA DAMPs are associated with inflammation and increased oxidative stress.^24^

Mitochondrial DAMPs are also increased in plasma in PMR vs. normal (*Figure 3C* and *D*), while glucose-6-phosphate dehydrogenase–dependent NADH production is decreased in PMR patients’ plasma vs. normal (*Figure 3E*) indicative of increased oxidative stress.^25^

### Glycogen accumulation in primary mitral regurgitation

#### Left ventricle

**Figure 4:**
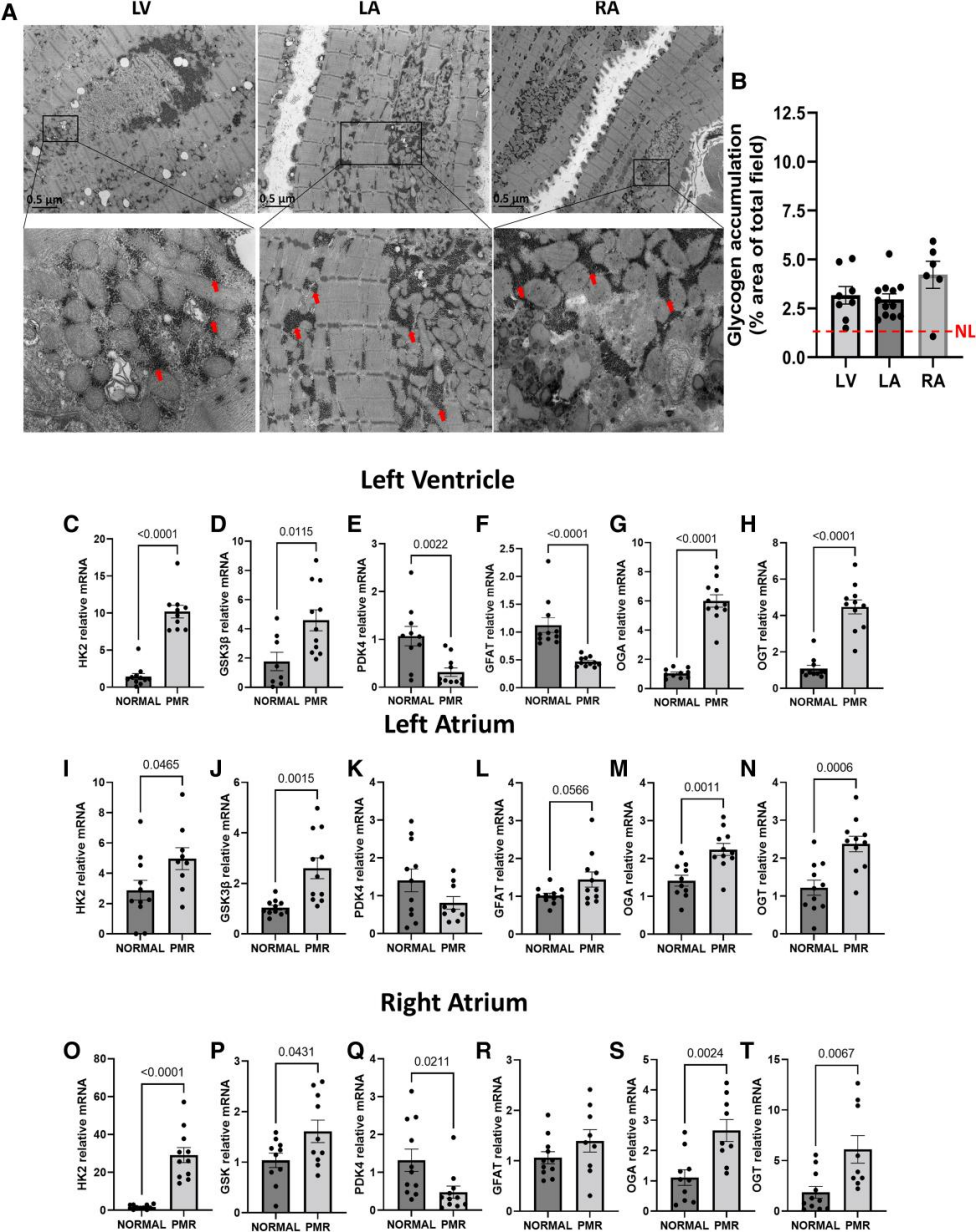
Glucose metabolism in patients with primary mitral regurgitation. (*A*) Representative transmission electron microscopy analysis of glycogen accumulation in primary mitral regurgitation heart biopsies (left ventricle, left atrial, and right atrial) (zoom areas are boxed, arrows show glycogen). Data were inferred from 10 fields for each heart region from 6 to 10 patients with primary mitral regurgitation. (*B*) Glycogen accumulation presented as % area of total field (magnification, ×5000). Data are presented as mean ± standard error. (*C–T*) Gene expression of glucose metabolism from the hearts of patients with normal and primary mitral regurgitation. Data were normalized to 18S and expressed as a fold change from normal hearts. The sample size for each measurement was *n* = 8–11 subjects/group. Data are presented as mean ± standard error.

Left ventricle TEM demonstrates increased glycogen in perinuclear, interfibrillar, and subsarcolemmal regions of the cardiomyocyte in addition to glycophagy (*Figure 4A*). Quantification is greater than the accepted normal of 2%. *Figure 4C* and *D* demonstrate increased hexokinase (HK) 2 and glycogen synthase kinase 3 beta (GSK3β) mRNA in the PMR LV vs. normal LV. *Figure 4E* shows a down-regulation of pyruvate dehydrogenase kinase (PDK) 4 mRNA in PMR vs. normal, while HK2 mRNA is increased along with (*Figure 4F* and *G*) the up-regulation of O-linked *N*-acetylglucosaminyl (O-GlcNAc) transferase (OGT), and O-GlcNAcase (OGA), which collectively indicate modification of the hexosamine biosynthetic pathway and O-GlcNAc pathway.

#### Left atrium and right atrium

Similar to the LV, there is increased glycogen accumulation in the PMR LA and RA (*Figure 4A* and *B*) along with an increase in HK2 and GSK3β mRNA in both LA and RA of PMR vs. normal (*Figure 4I*, *J* and *O*, *P*). Right atrium and LA also show a down-regulation of PDK4 mRNA (*Figure 4K* and *Q*) and an up-regulation of hexosamine biosynthesis pathway (HBP) genes: the rate-limiting enzyme in hexosamine biosynthesis and important pathway for cellular glucose sensing glutamine fructose-6-phosphate amidotransferase, OGA, and OGT (*Figure 4L–N* and *R–T*).

### Glucose metabolism in patients with primary mitral regurgitation

#### Left ventricle

**Figure 5:**
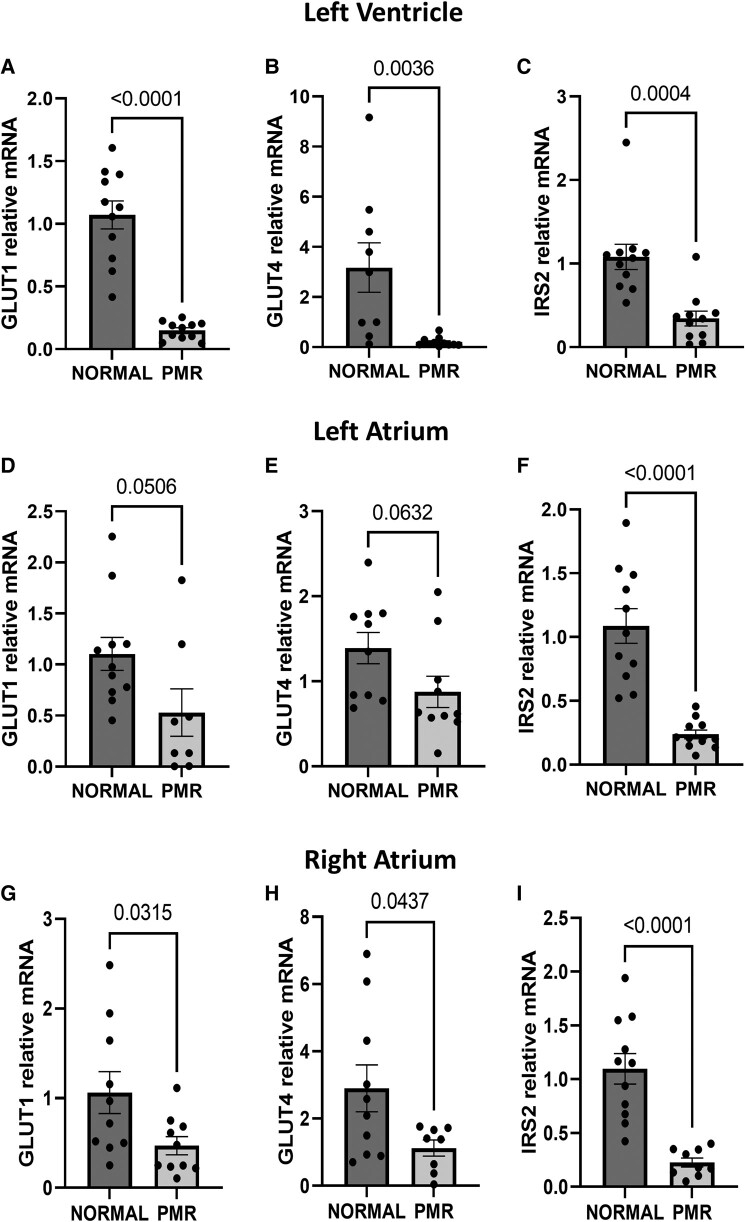
Glucose metabolism in patients with primary mitral regurgitation. (*A–I*) Gene expression of glucose metabolism from the hearts of patients with normal and primary mitral regurgitation hearts. Data were normalized to 18S and expressed as a fold change from normal hearts. The sample size for each measurement was *n* = 8–11.

There is a significant decrease in GLUT1, GLUT4, and insulin receptor substrate (IRS) 2 mRNA in the LV of the PMR patients vs. normal, indicative of decreased glucose uptake (*Figure 5A–C*).

#### Left atrium and right atrium

GLUT1, GLUT4, and IRS2 mRNA are also significantly decreased in RA PMR vs. normal; LA show a slight decrease in GLUT1 and GLUT4 mRNA, while IRS2 mRNA is significantly reduced (*Figure 5D–I*).

### Glucose uptake by 2-deoxy-2-[^18^F]fluoro-D-glucose-positron emission tomography /cardiac magnetic resonance in patients with primary mitral regurgitation

**Figure 6:**
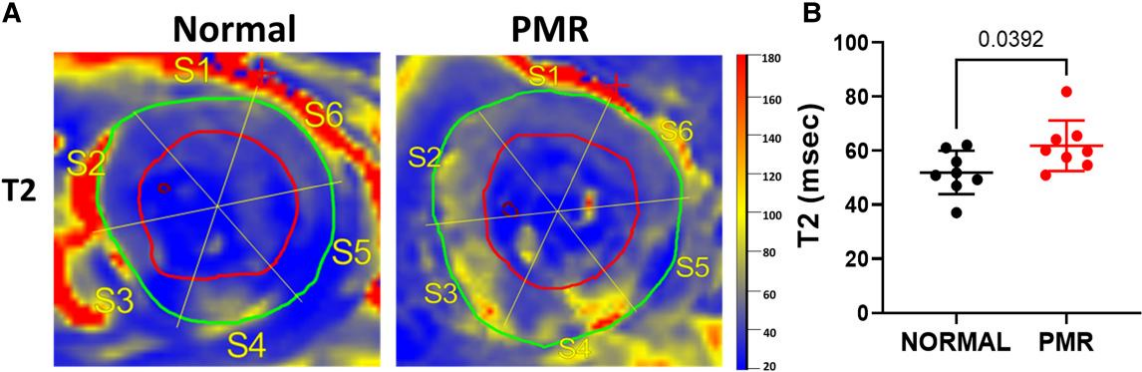
Inflammation in the left ventricle midwall in primary mitral regurgitation. (*A* and *B*) T2 heat map in the mid left ventricle of patients with normal and primary mitral regurgitation.

The reduction in the expression of myocardial glucose transporters and insulin receptor genes suggests that glucose uptake may be impaired in the PMR heart, which is a feature of insulin resistance. We thus assessed glucose uptake by [^18^F]FDG-PET/CMR in patients with a stable LVEF > 60% and moderate PMR. *Table 2* shows that LV end-diastolic volume, LV stroke volume, mean regurgitant volume, and LA maximum and minimum volumes are significantly greater in PMR vs. normal, while LV end-systolic volume and LVEF do not differ. Similarly, the mean decrease in total LA emptying fraction and LA expansion index do not achieve a statistical difference in PMR vs. normal. Left ventricle circumferential and radial strain rates are significantly greater in PMR vs. normal, while radial strain but not circumferential strain is greater in PMR vs. normal. Patients with PMR have increased T2 signal intensity vs. normals (*Figure 6*) indicative of increased inflammation and oedema.^26^

**Table 2:** Demographics and cardiac magnetic resonance indices from patients in whom 2-deoxy-2-[^18^F]fluoro-D-glucose-positron emission tomography/cardiac magnetic resonance was performed

	Normal	PMR	*P*-value
	(*n* = 8)	(*n* = 8)	
Age (years)	51 ± 9	63 ± 9	
Sex			
Female	5	4	
Male	3	4	
Race			
Black	1	1	
White	6	7	
Other	1		
Body mass index	28.0 ± 4.8	25.5 ± 4.6	
Hypertension		5 (63%)	
Medications			
None	7 (88%)	—	
Beta-blocker	—	5 (63%)	
ACE inhibitor	—	4 (50%)	
AT_1_RB	—	1 (13%)	
Calcium entry blocker	—	—	
Diuretics	—	5 (63%)	
Statins	—	4 (50%)	
Anti-arrhythmia	1 (12%)	—	
NYHA functional class			
I		4 (25%)	
II		3 (38%)	
III		1 (13%)	
Fasting glucose	84.5 ± 2.88	103.5 ± 11.81*	0.0147
HbA1C	5.25 ± 0.301	5.89 ± 0.57*	0.0161
Free fatty acids (µM)	154.09 ± 45.3	221.241 ± 97.6	0.0552
ApoA1 (mg/mL)	1.69 ± 0.89	1.559 ± 0.8	
Cardiac magnetic resonance			
LV end-diastolic volume (mL/m²)	63 ± 8	95 ± 20*	0.009
LV end-systolic volume (mL/m^2^)	26 ± 5	35 ± 12	0.06
LV stroke volume (mL/m^2^)	41 ± 7	60 ± 16*	0.008
LVEF (%)	61 ± 6	63 ± 10	0.7
Regurgitant volume (mL)	—	34 ± 16*	0.003
LV systolic circ. strain rate (1/s)	−0.79 ± 0.14	−1.05 ± 0.29*	0.04
LV systolic circ. strain (%)	−20.82 ± 2.48	−24.43 ± 5.26	0.1
LV systolic radial strain rate (1/s)	1.56 ± 0.32	2.25 ± 0.67*	0.01
LV systolic radial strain (%)	56.70 ± 11.88	84.55 ± 41.73*	0.03
LA maximum volume (mL/m^2^)	37 ± 12	91 ± 32*	<0.001
LA minimum volume (mL/m^2^)	19 ± 6	55 ± 24*	0.001
Total LA emptying fraction	47 ± 17	40 ± 15	0.4
LA expansion index	100 ± 49	77 ± 48	0.3

*P-value <0.05.

Values: mean ± SD.

LV, left ventricle; LA, left atrial; circ. strain, circumferential strain.

**Figure 7:**
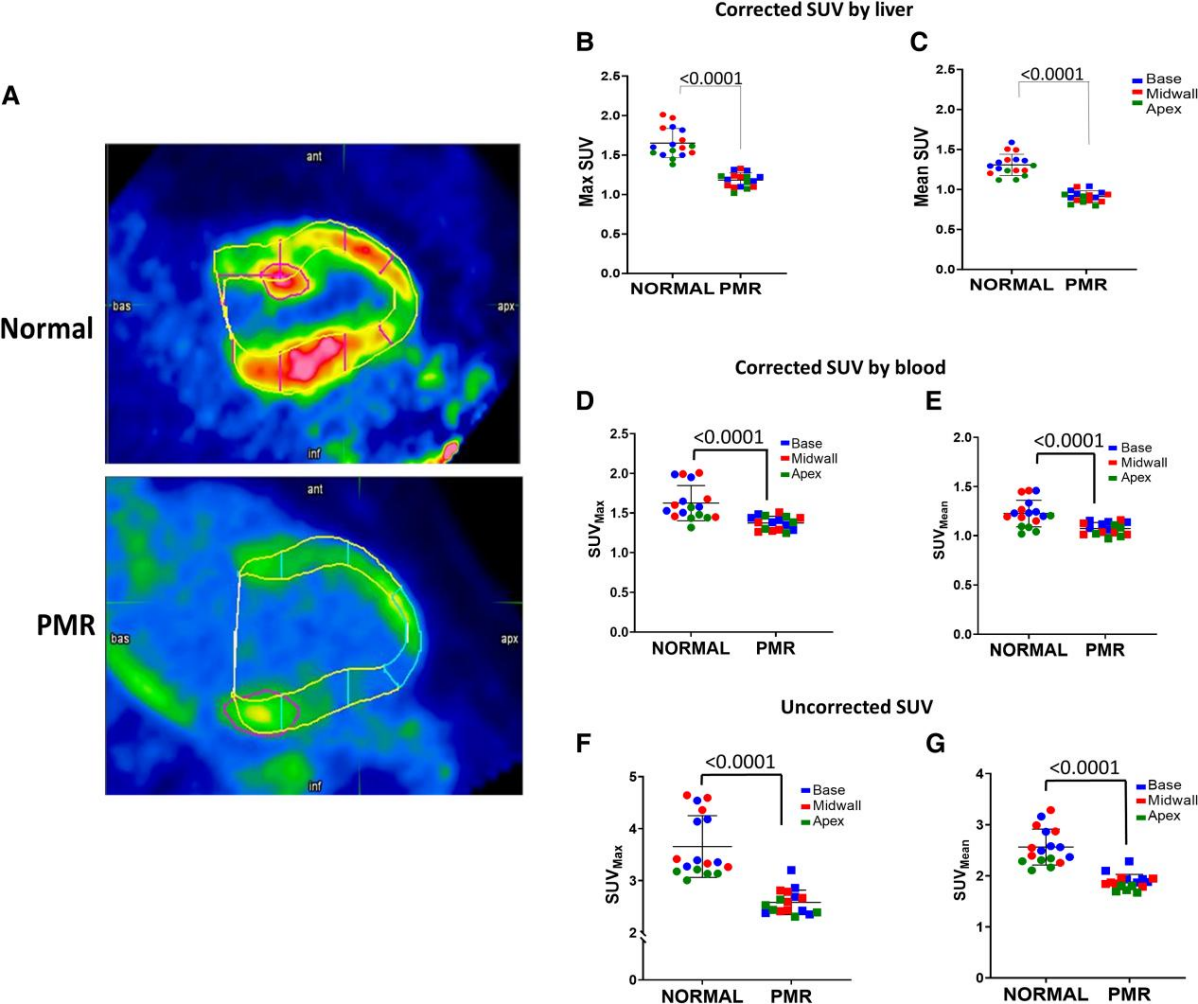
Myocardial glucose uptake. (*A*) Representative 2-deoxy-2-[^18^F]fluoro-D-glucose-positron emission tomography/MR myocardial uptake in patients with normal and primary mitral regurgitation, (focal point with high intensity= high uptake) and (low intensity = low uptake). (*B–G*) Seventeen segment analysis Max and mean myocardial standard uptake value (Max SUV and mean SUV) in patients with normal and primary mitral regurgitation, normalized by liver (*B* and *C*), blood (*D* and *E*), and uncorrected (*F* and *G*).

There is a significant decrease in SUV_mean_ and SUV_max_ [^18^F]FDG uptake in the LV base, midwall, and apex in PMR vs. normal when corrected by liver uptake, or when corrected for blood uptake and without correcting for other organ uptake (*Figure 7*). Patients with PMR present with haemoglobin A1C (HbA1c) >5.7%, suggestive of a pre-diabetic syndrome, and higher fasting plasma glucose and FFA levels compared with normal (*Table 2*).

## Discussion

In this study, we present a myocardial alteration in genes involved in fatty acid, glycogen, and glucose metabolism consistent with increased LDs, extensive glycogen accumulation, and mitochondria and myofibrillar breakdown. This striking collection of ultra-structural findings that are similar to end-stage heart failure reduced ejection fraction^27–29^ and diabetes^30–32^ are masked by a spuriously elevated LVEF due to ejection into the LA and neurohormonal activation.

### Lipid droplet accumulation

The striking increase in LDs in the RA, LA, and LV could be a consequence of increased NPY a co-transmitter released with norepinephrine.^33^ Increased catecholamine levels activate lipoprotein lipase, promote lipolysis, and increase circulating FFA levels.^34^ In the dog with experimentally induced PMR, there is increased cardiomyocyte lipid accumulation that inversely correlates with LV contractility.^35^ Lipid accumulation can also be caused by the decrease in CPT2 mRNA which controls oxidation of long-chain fatty acids and its decrease occurs in heart failure.^36^ The decrease in FASN in PMR hearts may be an adaptive response to prevent accumulation of free cytosolic lipids, reflected in a significant reduction of FFA PCF vs. plasma levels. However, FASN knockout mice die within 1 day of trans-aortic constriction due to LV failure and hyper-activation of Ca^2+^/calmodulin-dependent protein kinase II signalling.^37^ Meanwhile, overexpression of FASN develop heart failure rescued by GRK2 inhibition.^38^ These findings reinforce the connection between prolonged G-protein-coupled activation and fatty acid metabolism.

### Glycogen accumulation

The decrease in GLUT1 and GLUT4 mRNA and ISR2 can result in shunting of intracellular glucose storage to glycogen.^39,40^ There is an accumulation of glycogen across RA, LA, and LV in addition to glycogen within double-membrane phagosome structures indicative of autophagy-mediated degradation of excessive glycogen.^41^ Excessive glycogen is consistent with the increase in HK2 and GSK3β mRNA. Besides its role in glucose metabolism, GSK3β is also associated with lipid accumulation, cardiac oxidative stress, and inflammation.^40^ Moreover, the activation of GSK3β in combination with down-regulation of IRS2 further lower cellular glucose uptake.^39,40^ The decrease in PDK4 mRNA may be an adaptive response to stimulate glucose oxidation and improve cardiac glucose uptake,^42^ while increased HK2 mRNA is associated with up-regulation of the hexosamine biosynthetic pathway and O-GlcNAcylation of proteins, which leads to mitochondrial dysfunction.^43^

### Mitochondrial damage

The increase in PCF mtDAMPs over simultaneous plasma levels is consistent with the extensive mitochondrial cristae lysis across RA, LA, and LV. Mitochondrial DAMPs activate a pro-inflammatory response,^44^ which is consistent with increased T2 that is indicative of inflammation and oedema^26^ and significant elevation of cytokines, macrophage markers, and matrix metalloproteinases(MMP) in the PCF over plasma in PMR.^23^ Despite normal epicardial coronary arteries in PMR, an increased inflammatory state combined with haemodynamic overload and wall stress can lead to a microcirculatory deficiency, hypoxia, and mitochondrial dysfunction. Indeed, there is an inverse relationship between the decrease in phosphocreatine to adenosine triphosphate (ATP) ratio and LV dilatation consistent with a mitochondrial bioenergetic defect in patients with PMR.^45^

### Insulin receptor and glucose transporter type 1 and glucose transporter type 4 down-regulation

Chronic neurohormonal activation, in particular increased β_1_AR activation, in addition to haemodynamic overload, can account for the decrease in GLUT4 and GLUT1 mRNA and ISR2 expression in LV and LA. The RA is subjected to increased pulmonary pressure and moderate tricucuspid regurgitation in all but two patients with PMR. Increased circulating and PCF NPY levels suggest a connection to an increase in myocardial sympathetic activity. In patients with a moderately reduced LVEF, the decrease in pre-synaptic noradrenaline re-uptake correlated with myocardial glucose uptake using [^18^F]FDG, suggesting that the higher neurotransmitter βAR activation leads to decreased glucose uptake.^15^ Myocardial glucose uptake is also positively regulated by ApoA1,^46,47^ which is significantly decreased in PCF vs. plasma in our patients with PMR. In a proteomics study of patients with PMR, reduced ApoA1 was predictive of the severity of mitral regurgitation.^48^ Taken together, these studies support the potential utility of metabolic imaging and biomarkers in the staging of PMR.

#### 2-Deoxy-2-[^18^F]fluoro-D-glucose uptake

The decrease in GLUT1, GLUT4, and IRS2 mRNA in patients with surgical PMR led us to examine [^18^F]FDG uptake in patients with stable moderate PMR. Previous studies have reported a localized increase in [^18^F]FDG in the peri-papillary muscle area in patients with PMR, which has been linked to inflammation and ventricular arrhythmias.^49,50^ Our study is the first to report a global decrease in LV [^18^F]FDG uptake in PMR vs. normal subjects, together with a higher fasting blood glucose and HbA1c levels of 5.7–6.4. Reducing glucose transport may be an initial adaptive response to protect the heart from a glucose overload. Other findings from our biopsies, such as up-regulation of the HK2, GSK3β, and HBP and glycogen storage, support this hypothesis. Further studies must establish whether this metabolic alteration precedes a decrease in the LVEF below 60% which has a higher mortality with surgery.^51^

### Study limitations and perspectives

A limitation of the current study is that the metabolic gene profile and cardiomyocyte ultra-structure are a single snapshot in time of surgical patients with a more severe PMR. As such, this cannot address whether a picture of insulin resistance and cardiomyocyte ultra-structural damage is an adaptation or maladaptation in PMR. This calls for a larger scale investigation into the role of biomarkers and [^18^F]FDG-PET imaging, especially in asymptomatic PMR, to determine whether such a metabolic derangement may determine the timing of surgical intervention.

### Clinical perspectives

- The PMR heart has lipid and glycogen accumulation, mitochondrial damage, and a metabolic gene profile consistent with severely impaired lipid and glucose metabolism.
- Patients with PMR have decreased myocardial [^18^F]FDG uptake as well as increased circulating FFAs, HbA1c levels, and mtDNA DAMPs.
- Reduced myocardial glucose uptake in patients with moderate PMR suggests that metabolic inflexibility is an early event and precedes Heart Failure (HF) symptoms.
- Given its heart failure risk, decreased glucose uptake, and circulating FFAs, ApoA1 and HbA1c levels may be attractive markers for myocardial metabolic inflexibility assessment to improve timing for surgical intervention in PMR.

## Supplementary Material

oeae034_Supplementary_Data

## Data Availability

Data can be shared upon request.

## Lead author biography

Dr Mariame Selma Kane, PhD, is a Health Systems Specialist at the Birmingham Veterans Affairs (VA) Health Care System, recipient of the 2023 VA Southeast Network (VISN7), Research Development Award (VISN7), and 2024 Diversity Supplement under mentor Dr Louis J. Dell’Italia, MD. Her research focuses on the interplay between cardiovascular disease, mitochondrial dysfunction, and metabolic inflexibility. Dr Kane also studies the genetic epidemiology and molecular mechanisms of heart failure in racially disparate populations. The work presented here follows from a pre-clinical model of PMR, on how mitochondrial haplotype affects cardiomyocyte mitochondrial ultra-structure and transcriptome response to a volume overload.
