# Supplementary figures and images for: Gene expression and ultra-structural evidence for metabolic derangement in the primary mitral regurgitation heart
Source: Eur Heart J Open. 2024 May 1;4(3):oeae034. doi: 10.1093/ehjopen/oeae034 (PMC11157345; doi:10.1093/ehjopen/oeae034)

**Supplemental Material**

**Supplementa1 Table 1: List of Taqman probe and primer sets**


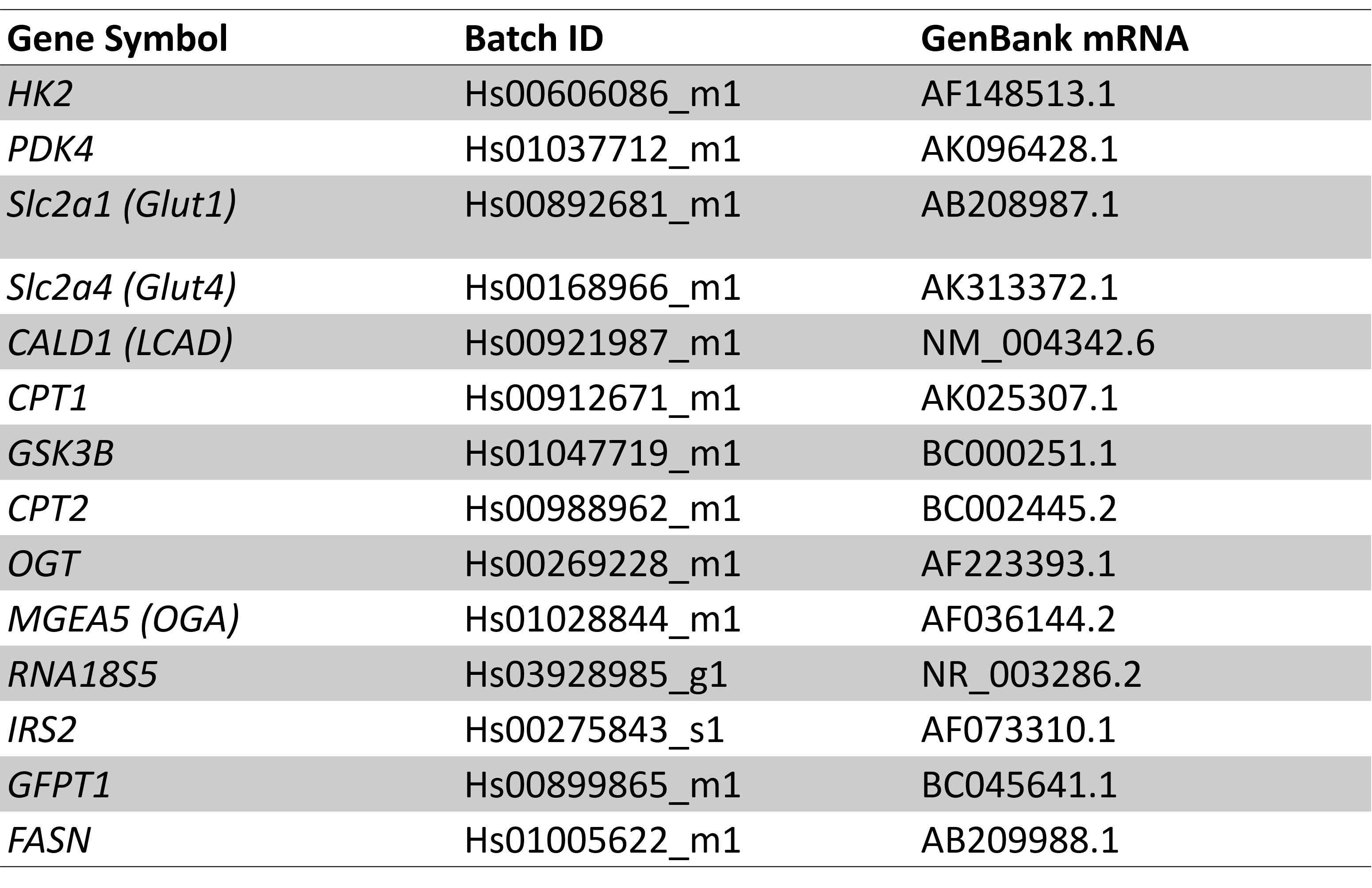

Supplement: oeae034_Supplementary_Data [file oeae034_supplementary_data.docx]
